# Prevalence of unrecognized myocardial infarction in a low–intermediate risk asymptomatic cohort and its relation to systemic atherosclerosis

**DOI:** 10.1093/ehjci/jew155

**Published:** 2016-08-22

**Authors:** Jonathan R. Weir-McCall, Kerrie Fitzgerald, Carla J. Papagiorcopulo, Stephen J. Gandy, Matthew Lambert, Jill J.F. Belch, Ian Cavin, Roberta Littleford, Jennifer A. Macfarlane, Shona Z. Matthew, R. Stephen Nicholas, Allan D. Struthers, Frank M. Sullivan, Shelley A. Waugh, Richard D. White, J. Graeme Houston

**Affiliations:** 1 Department of Cardiovascular and Diabetes Medicine, College of Medicine, Ninewells Hospital, University of Dundee, Level 7, Dundee DD1 9SY, UK; 2 NHS Tayside Medical Physics, Ninewells Hospital, Dundee, UK; 3 Department of Research and Innovation, North York General Hospital, University of Toronto, Toronto, UK; 4 Department of Clinical Radiology, University Hospital of Wales, Cardiff, UK

**Keywords:** myocardial infarction, whole-body MRI, cardiac magnetic resonance, atherosclerosis

## Abstract

**Aims:**

Unrecognized myocardial infarctions (UMIs) have been described in 19–30% of the general population using late gadolinium enhancement (LGE) on cardiac magnetic resonance. However, these studies have focused on an unselected cohort including those with known cardiovascular disease (CVD). The aim of the current study was to ascertain the prevalence of UMIs in a non-high-risk population using magnetic resonance imaging (MRI).

**Methods and results:**

A total of 5000 volunteers aged >40 years with no history of CVD and a 10-year risk of CVD of <20%, as assessed by the ATP-III risk score, were recruited to the Tayside Screening for Cardiac Events study. Those with a B-type natriuretic peptide (BNP) level greater than their gender-specific median were invited for a whole-body MR angiogram and cardiac MR including LGE assessment. LGE was classed as absent, UMI, or non-specific. A total of 1529 volunteers completed the imaging study; of these, 53 (3.6%) were excluded because of either missing data or inadequate LGE image quality. Ten of the remaining 1476 (0.67%) displayed LGE. Of these, three (0.2%) were consistent with UMI, whereas seven were non-specific occurring in the mid-myocardium (*n* = 4), epicardium (*n* = 1), or right ventricular insertion points (*n* = 2). Those with UMI had a significantly higher BNP [median 116 (range 31–133) vs. 22.6 (5–175) pg/mL, *P* = 0.015], lower ejection fraction [54.6 (36–62) vs. 68.9 (38–89)%, *P* = 0.007], and larger end-systolic volume [36.3 (27–61) vs. 21.7 (5–65) mL/m^2^, *P* = 0.014]. Those with non-specific LGE had lower diastolic blood pressure [68 (54–70) vs. 72 (46–98) mmHg, *P* = 0.013] but no differences in their cardiac function.

**Conclusion:**

Despite previous reports describing high prevalence of UMI in older populations, in a predominantly middle-aged cohort, those who are of intermediate or low cardiovascular risk have a very low risk of having an unrecognized myocardial infarct.

## Introduction

Unrecognized myocardial infarcts (UMIs) have been described in 19–44% of the general population, with prevalence increasing by 10% per decade and their presence associated with a similar or worse prognosis than recognized myocardial infarcts (RMIs).^1,2^ The majority of population-based studies have focused on the use of electrocardiogram (ECG) for the detection of UMI; however, not all infarcts result in pathological Q waves.^3^ Late gadolinium enhancement (LGE) on cardiac magnetic resonance (CMR) imaging has become the clinical gold standard for the detection of myocardial scarring, with a significantly higher detection rate of UMIs than using ECG alone.^4^ These UMIs detected on CMR have significant clinical implications, with those with evidence of myocardial scarring in the absence of clinically apparent prior infarct more likely to have chest pain and poorer left ventricular (LV) function and suffer from a greater number of major adverse cardiovascular events.^4,5^

Earlier studies have described the prevalence of UMIs in elderly populations at high risk of cardiovascular disease (CVD). These studies, by including those with known CVD, have thereby conflated the prevalence of UMIs in the general population.^4,6^ The prevalence of UMIs in a population without prior cardiovascular events and who are not at high risk has not been previously undertaken. Furthermore, a prior study of 75-year-olds has suggested that UMIs may not be associated with traditional CVD risk factors.^7^ Thus, the identification of a cohort considered as low or intermediate risk that have suffered from UMIs may provide insight into novel predisposing aetiological factors.

The aim of this study was to investigate the prevalence of UMIs in a large, non-high-risk, asymptomatic cohort, assessed with magnetic resonance imaging (MRI), and the association between UMIs and risk markers for CVD.

## Methods

### Study population

Following local ethics committee approval, a cohort of *n* = 1651 volunteers were recruited to the imaging arm of the Tayside Screening for Cardiovascular Events (TASCFORCE) study. Volunteers were recruited to the study via general practitioner (GP) surgeries, advertising on radio and via leaflets distributed at local public events and via large local employers. They were enrolled into TASCFORCE if they met the inclusion criteria that they (i) were above the age of 40 years, (ii) were free from CVD or other indication for statin therapy as recommended by the Scottish Intercollegiate Guidelines Network (SIGN) report 97 (www.sign.ac.uk) for ‘Risk Estimation and the Prevention of Cardiovascular Disease’ published in February 2007, and (iii) had a 10-year risk of coronary heart disease <20% as predicted by the Adult Treatment Panel III (ATP-III) algorithm.^8^ Exclusion criteria included the following: (i) pregnancy; (ii) known primary muscle disease; (iii) known atherosclerotic disease—including angina, previous myocardial infarction, peripheral arterial disease, amputation, previous revascularization surgery, hypertension, heart failure, or cerebrovascular event; (iv) known diabetes; (v) active liver disease; (vi) other known illness or contraindication to MRI; (vii) participation in a clinical trial; (viii) inability to give informed consent; (ix) known alcohol abuse; and (x) a blood pressure of >145/95mmHg. Participants who had a serum B-type natriuretic peptide (BNP) level greater than their gender-specific median (7.5 pg/mL in men, 15.3 pg/mL in women), indicating possible non-specific stress on the cardiovascular system, were invited for whole-body MRI angiography. Except for 14 men and 2 women, all participants had a BNP level of <100 pg/mL, which is the threshold used for the diagnosis of heart failure, and therefore the vast majority of participants had a BNP in a ‘normal’ range. Of 1651 volunteers, 122 were excluded because of claustrophobia or MR safety concerns, with 1529 (91.4%; 931 females and 577 males, mean age 54 years, range 40–83 years) completing their imaging.

### Image acquisition

The MRI protocol has been described in detail elsewhere,^9^ but in brief, imaging was performed using a 3 T Magnetom Trio Scanner (Siemens, Erlangen, Germany). Whole-body MR angiography was performed using a dual bolus injection technique with the CMR CINEs performed before the first contrast injection and the LGE sequences performed between the first and second contrast bolus injections. For CMR, a body matrix radiofrequency coil (6 elements) was used in combination with a spine array (up to 24 elements).

ECG-gated, segmented, breath-hold, cinematic (CINE) TrueFISP images were acquired from the atrioventricular ring to the LV apex using 2D ECG-gated, breath-hold, segmented (CINE) TrueFISP sequence with retrospective gating. Retrospective ECG gating was used, with 25 cardiac phases reconstructed (25 lines per segment) and 2 image slices acquired per breath-hold. Parallel imaging was also implemented [integrated parallel acquisition technique (iPAT) ×2], resulting in a scan time of <15 s per breath-hold. An ECG-gated, segmented, breath-hold, CINE 2D turbo Inversion Recovery ‘TI-scout’ sequence was implemented (in a central short-axis orientation) 8–10 min after the injection of 10 mL of 0.5 mmol/mL gadoteric acid. At a median of 11 min post-contrast (range 9–16 min), a short-axis stack of ECG-gated, segmented, 2D phase-sensitive inversion recovery images were acquired.^10^

### Image analysis

LV mass and volume and whole-body atheroma burden quantification were performed as previously described.^9^

Analysis of the LGE sequences was performed offline on a diagnostic PACS radiological workstation (Kodak Carestream PACS Client Suite Version 10.1 sp1, Rochester, NY, USA) by one of the two experienced observers [one with 5 years (J.R.W.M.) and one with 15 years of experience (J.G.H.)] independently, with each scan being recorded as either scar-positive or scar-negative. All positive scans were then classified by consensus opinion with LGE classified as being sub-endocardial, mid-myocardial, epicardial, or other. Thickness was scored as <50%, >50%, or transmural. Sub-endocardial and transmural scarring were classed as UMI. The location and extent were scored using the American Heart Association (AHA) 17 segment model.^11^

The whole-body MRA analysis was performed as previously described.^9^ In brief, the arterial tree was divided into 31 segments with each scored according to the degree of narrowing of the lumen diameter, with stenosis graded at the narrowest part of the vessel. Each vessel was scored from 0 to 4, where 0, segment with no stenosis; 1, <50% stenosis; 2, 51–70% stenosis; 3, 71–99% stenosis; and 4, vessel occlusion. The ‘standardized atheroma score’ (SAS) was calculated by summing each individual segment's stenosis score and divided by the number of diagnostic segments (*n*) before dividing by 4 that is the maximum potential score [Eq. (1)]:

(1) $$\text{SAS}=\left[\frac{\Sigma \;\text{MRA score/}n}{4}\right]\times 100$$

### Statistical analysis

Data are expressed as mean ± standard deviation (SD) for normally distributed continuous variables, median (range) for non-normally distributed continuous and ordinal variables, and *n* (%) for nominal variables. To test the null hypothesis that samples originate from the same distribution, an independent sample Kruskal–Wallis test was used, with *post hoc* Mann–Whitney *U* test to further evaluate between group differences when a significant difference was observed from the Kruskal–Wallis test. Fisher's exact test was used to compare nominal data. All data were analysed using SPSS statistical package (version 21.0, IBM SPSS, Chicago, IL, USA). Significance was assumed when *P* < 0.05.

## Results

Fifty-three of the 1529 (3.6%) CMRs were excluded because of either missing data or inadequate LGE image quality. Ten of the remaining 1476 (0.67%) displayed delayed myocardial enhancement, of which 90% were female (age 54 ± 8 years). Of these, three (0.2%) demonstrated sub-endocardial enhancement in a pattern consistent with UMIs. Of these three,

**Figure 1:**
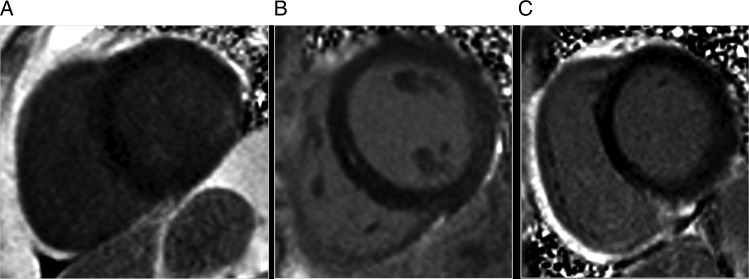
Examples of myocardial enhancement patterns observed. (*A*) Sub-endocardial enhancement in the inferolateral wall of the basal left ventricle consistent with a myocardial infarct. (*B*) Mid-myocardial enhancement. (*C*) RV insertion point enhancement.

- one involved eight AHA segments in the left anterior descending (LAD) territory, with full thickness involvement of the apical segments and <50% thickness involvement in the mid-cavity segments, and an ejection fraction of 36.3%;
- the second involved six segments in the LAD territory, with full thickness involvement of the apical segments and <50% thickness involvement in the mid-cavity segments, associated with regional hypokinesia, but a preserved ejection fraction of 55%;
- the final infarct involved two segments in the inferolateral wall basally, with <50% myocardial thickness scarring, associated with regional wall motion abnormality but a preserved ejection fraction (see *Figure 1*).

Of the remaining seven (0.47%),

- one demonstrated epicardial enhancement involving one AHA segment;
- four had a mid-myocardial pattern of enhancement, all four of which involved a single AHA segment;
- two had right ventricular septal insertion point enhancement each involving one AHA segment.

None of these were associated with regional wall motion abnormalities.

Of the three patients with UMI pattern LGE, only one had clinical symptoms describing shortness of breath on exertion, which had been labelled by their GP as chronic obstructive pulmonary disease because of a history of smoking and an obstructive pattern on spirometry, but on review was felt to more likely be owing to their systolic impairment. Single-photon emission computed tomography (SPECT) study confirmed a large irreversible perfusion defect in the LAD territory. Neither of the other two with UMI exhibited any chest pain or shortness of breath. Those with atypical LGE patterns were asymptomatic, with no prior recalled episodes of significant chest pain.

The demographic data are described in *Table 1*. Those with UMIs had a significantly higher BNP [116 (31–133) vs. 22.6 (5–175) pg/mL, *P* = 0.015] compared with the group without the evidence of LGE but were otherwise similar in their baseline measures. Those with non-specific scarring had lower diastolic blood pressure [68 (54–70) vs. 72 (46–98)mmHg, *P* = 0.013], but were otherwise similar to those without scarring. Imaging findings of the two groups are described in *Table 2*. Compared with those without scarring, those with UMIs had significantly lower ejection fractions [54.6 (36–62) vs. 68.9 (38–89)%, *P* = 0.007] and higher end-systolic volumes [36.3 (27–61) vs. 21.7 (5–65) mL/m^2^, *P* = 0.014], whereas those with non-specific LGE demonstrated no significant differences in cardiac mass, volume, or function. Neither group demonstrated a significantly higher atheroma burden than those with no scarring (*P* = 0.27).

**Table 1:** Comparison of cohort characteristics between those with and without late gadolinium enhancement

	No LGE	UMI	Non-specific LGE	*P*
*n* (%)	1463 (99.1)	3 (0.2)	7 (0.5)	
Male (%)	561 (38)	0 (0)	1 (14)	0.26
Age (years)	53.4 (40–80)	62 (53–66)	58.1 (50–67)	0.13
Pulse	62 (35–92)	67 (61–82)	62 (54–70)	0.44
Systolic BP	122 (80–145)	118 (104–134)	128 (102–140)	0.71
Diastolic BP	72 (46–98)	64 (58–76)	68 (58–70)	**0.018**
Total cholesterol	5.4 (2.7–9.5)	5.4 (5.2–6.4)	5.4 (4.6–7.2)	0.87
LDL-cholesterol (mmol/L)	3.4 (0.9–6.8)	3.3 (3.2–3.9)	3.2 (2.3–4.7)	0.96
HDL-cholesterol (mmol/L)	1.4 (0.4–2.6)	1.8 (1.3–1.8)	1.5 (0.9–2.3)	0.94
Triglycerides (mmol/L)	1.3 (0.5–7)	1.6 (0.5–1.7)	1.1 (0.5–2.5)	0.37
Random glucose	5.1 (3–11)	5.3 (5–6)	6.2 (5–8)	0.11
ASSIGN risk score	7.4 (0.9–48)	10.3 (10–13)	6 (3–17)	0.46
BMI (kg/m^2^)	26.1 (17–43)	27.6 (25–29)	22.9 (21–31)	0.09
Current/ex-smoker (%)	552 (38)	1 (33)	3 (43)	1
Smoking pack years	0 (0–105)	0 (0–30)	0 (0–57)	0.91
FH of CVD (%)	377 (26)	1 (33)	0 (0)	0.2
BNP (pg/mL)	22.7 (5–175)	116 (31–133)	22.6 (10–62)	**0.05**

ASSIGN, ASsessing cardiovascular risk using SIGN guidelines; BMI, body mass index; BNP, brain natriuretic peptide; BP, blood pressure; CVD, cardiovascular disease; FH, family history; HDL, high density lipoprotein; LDL, low density lipoprotein; LGE, late gadolinium enhancement; UMI, unrecognized myocardial infarction.

Bold indicates statistical significance.

**Table 2:** Comparison of left ventricular measures and whole-body atheroma burden between the groups

	No LGE	UMI	Non-specific LGE	*P*
LVM^a^ (g/m^2^)	53.6 (26–109)	64.1 (59–67)	47.7 (40–60)	0.08
LVEDV^a^ (mL/m^2^)	70.5 (38–140)	79.9 (72–96)	69.6 (61–81)	0.23
LVESV^a^ (mL/m^2^)	21.7 (5–66)	36.3 (27–61)	19.9 (13–28)	**0.033**
LVSV^a^ (mL/m^2^)	48.3 (24–92)	43.7 (35–45)	49.7 (40–54)	0.20
LVEF (%)	68.9 (38–89)	54.7 (36–62)	71.4 (65–78)	**0.019**
LVMVR (g/mL)	0.76 (0.4–2.1)	0.74 (0.7–0.9)	0.70 (0.6–0.8)	0.30
SAS	0 (0–19.6)	4.2 (0–9)	0 (0–2)	0.27

LGE, late gadolinium enhancement; LVM, left ventricular mass; EDV, end diastolic volume; ESV, end-systolic volume; EF, ejection fraction; SV, stroke volume; LVMVR, left ventricular mass volume ratio; SAS, standardized atheroma score; UMI, unrecognized myocardial infarction.

^a^Normalized to body surface area.

## Discussion

In this study, we have demonstrated that those who are of middle-age with an intermediate or low cardiovascular risk have a very low risk of UMIs. The prevalence of UMIs of 0.2% in our study is similar to the 0.34% reported by Goehde *et al.*,^12^ although this prior study had focused on a younger age group than the current study with a mean age of 49.7 compared with our 54.2 years, 15.8% of their population being under 40 with under 40s excluded from recruitment within our study and finally only 2.6% of their population were over 65 compared with our 10.5%. This is important as two prior studies focusing on 70- and 75-year-olds, respectively, described a prevalence of 19.8% in the 70-year-old cohort and 30% in the 75-year-olds, consistent with a strong age-related association.^4,13^ The same group also demonstrated a lack of association between the presence of UMIs and traditional risk factors, carotid intima media thickness, high-sensitivity C-reactive protein, or whole-body atheroma burden.^7^ Although the average age of our study was significantly lower than that observed in these two prior studies, there were 331 participants over 60 years of age and 63 over the age of 70; thus, a significantly higher incidence of UMI would have been expected in our cohort on the basis of these prior studies. This suggests that the lack of observed differences between the population with and without UMI in these previous studies may have simply been due to being underpowered to detect differences, because when we have excluded those considered at high risk of CVD (calculated using standard risk factor measurements), the prevalence of UMIs is vanishingly low. A previous study by Schelbert *et al*. reported an incidence of UMIs of 17%, but this was again a much older population, including high-risk participants and those with known coronary artery disease.^6^ Contrary to Barbier's work and supported by our own study, they found a significantly greater prevalence of traditional CVD risk factors in those with UMIs compared with those without. They also found the incidence to be significantly higher in those with diabetes. Indeed, in comparison to our current study, a healthy population with diabetes and similar demographic characteristics as our group demonstrated a prevalence of 6% UMIs.^14^

Knowledge of the prevalence and population likely to have UMIs is important for several reasons. First and most importantly is their significant prognostic implications, with those with UMIs at markedly increased risk of future cardiovascular events.^1,6^ Indeed in our study, UMIs had significant functional implications, with these associated with reduced ejection fraction, dilation of the LV cavity, and an elevated BNP. Those with UMIs have been shown to have a high prevalence of significant coronary stenosis, both globally within the coronary vessels and focally upstream from the lesion, particularly in UMIs occurring in the LAD territory.^15,16^ There are promising data showing the benefits of intervening in silent ischaemia,^17^ thus this population may benefit from more aggressive management. However, prior studies have focused on SPECT assessment, and thus further work on those with asymptomatic UMIs recognized by CMR is still required. Second, if a high prevalence of UMIs was to be observed in a young cohort, it would suggest significant missed opportunity for primary prevention. Our finding of a very low rate of UMIs in a predominantly middle-aged population without the high risk of CVD provides reassurance that this is not the case and that prevention efforts need not occur at excessively young ages, instead being better focused on middle and older ages.^18^

More prevalent than UMIs in our current cohort, but still uncommon affecting only 0.47% of the group, was LGE in a pattern not typical of myocardial infarction. The most common of these was mid-myocardial enhancement, which is non-specific and can be seen in hypertensive cardiomyopathy, infiltrative cardiomyopathies, and myocarditis.^19^ Given that those with high blood pressure were excluded from the study and that the LV mass was not significantly elevated in these cases, the former two are less likely, leaving prior undiagnosed myocarditis as a possible source of this myocardial enhancement. In these participants with possible subclinical events, the lack of difference in ventricular function or volumes is reassuring that there do not appear to be any significant downstream sequelae of these scars. This is in agreement with recent work showing the lack of significant impact on prognosis between those with and without LGE in patients with clinically apparent myocarditis.^20,21^ The finding of a lower diastolic blood pressure in the cohort with non-specific LGE compared with the cohort without any LGE is uncertain but may be a manifestation of the high prevalence of females in the non-specific group, or possibly erroneous because of the small group size.

There are several weaknesses in the current study. We acquired the LGE following a single-dose contrast injection, whereas clinical routine practice is for this to be performed following double dose. However, at 3 T, contrast exerts a greater effect on T1 relaxation, and single-dose LGE has been proved to be equivalent with double dose imaging for scar detection.^22^ Our LGE imaging was acquired only in the short-axis plane, thus apical lesions that are better appreciated on long-axis views may have been missed, although previous reports have shown this to be an uncommon location for UMIs.^4^ In addition, the lack of a second plane for correlation may have led to under-reporting of areas of indeterminate signal, potentially underestimating the true prevalence. The numbers of both the UMIs and non-specific LGE were small meaning only limited conclusions can be drawn from inter-group data comparison. Additionally, this was a single-centre study on a predominantly white population, thereby limiting the generalizability of our findings to other groups. Participants were recruited based on an old threshold for the diagnosis of hypertension (145/90mmHg) and using the ATP-III criteria, both of which were the reference standard at the point of study design, but have been superseded by more up-to-date risk criteria. Thus, our healthy cohort included some participants with what would now be considered hypertension. However, this would be expected to bias the results in favour of a higher rate of UMIs; thus, our findings of a low prevalence in this cohort remain valid. For the purposes of this paper for comparing risk between cohorts, we have instead adopted the ASSIGN score, as this has the greatest evidence basis in a Scottish cohort.^23^

## Conclusion

Despite previous reports describing high prevalence of UMI in older populations, in a predominantly middle-aged cohort, those who are of intermediate or low cardiovascular risk have a very low risk of having an UMI.

## Funding

The study was funded by the Souter Charitable Foundation and the Chest, Heart and Stroke Scotland Charity. J.R.W.M. is supported by the Wellcome Trust through the Scottish Translational Medicine and Therapeutics Initiative (grant no. WT 085664) in the form of a clinical research fellowship. Neither group had any role in the study design, collection, analysis, and interpretation of data; in the writing of the manuscript; or in the decision to submit the manuscript for publication.

**Conflict of interest:** J.R.W.M. has received monies from Guerbet for attending symposia and for running educational meetings. J.G.H. is Director and Shareholder of Vascular Flow Technologies Ltd and has received research funds from Guerbet.
